# Prompt Determination of the Mechanical Properties of Industrial Polypropylene Sandwich Pipes

**DOI:** 10.3390/ma14092128

**Published:** 2021-04-22

**Authors:** Sergejs Vidinejevs, Rafal Chatys, Andrey Aniskevich, Krzysztof Jamroziak

**Affiliations:** 1 Institute for Mechanics of Materials, University of Latvia, Jelgavas iela 3, LV-1004 Riga, Latvia; sergejs.vidinejevs@pmi.lu.lv (S.V.); andrey.aniskevich@pmi.lu.lv (A.A.); 2 Department of Computer Techniques and Armament, Faculty of Mechatronics and Machine Building, Kielce University of Technology, Tysiąclecia Państwa Polskiego 7 Str., 25-314 Kielce, Poland; chatys@tu.kielce.pl; 3 Department of Mechanics, Materials and Biomedical Engineering, Wroclaw University of Science and Technology, Smoluchowskiego 25 Str., 50-370 Wroclaw, Poland

**Keywords:** polypropylene pipes, multilayer pipes, ultimate strength, experimental tests, FE analysis

## Abstract

A simple and prompt method to determine the mechanical properties of industrial multilayer extrusion polypropylene pipes for a gravity sewer network is suggested. The engineering formulas included for calculating the permissible thickness and relative position of a foam core in the pipes are based on a linear-elastic approximation and the rule of mixtures. The applicability of the approximation was justified experimentally during investigation of the effective tensile characteristics of single- and multilayer pipes and each layer specimen by using traditional tests and finite-element calculations. The results obtained were used to formulate engineering recommendations for calculations of this type.

## 1. Introduction

Polypropylene (PP) polymers have several advantages over other thermoplastics (polystyrene and polyethylene). PP can be used at increased operating temperatures; it has good stiffness and impact resistance, excellent chemical resistance, and long service life [1,2,3]. PP pipes occupy a prominent place in the list of structural elements produced industrially. Estimates indicate that up to 10% of 53 million tons of the total PP consumed was used in the form of pipes in the world in 2015 [4]. The advantages of PP pipes contributing to the conquest of the market include easy methods of their processing and installation, low cost, ease of disposal and an acceptable index of influence on the sustainability index. PP products are of universal demand in comparison with many other polymers [5,6], and PP composites are also used to reinforce steel pipes [7,8].

There is a tendency in the production of PP pipes to replace continuous single-layer pipes with multilayer pipes [9,10], whose outer and inner layers are made of high-strength PP and an intermediate layer (core)—of PP foam.

Thermoplastic foams have a cellular structure created by an expanding blowing agent (usually a gaseous phase) dispersed in the polymer melt. Due to their increased stiffness-to-weight ratios, the foams reduce the amount of PP used and, accordingly, diminish the cost of finished products. Efforts to improve the foaming by modifying or developing new PP resins, as well as to improve extrusion processes, leading to better material properties, are described in [1,11,12,13,14,15,16].

The protection of connections and cables is an essential element in various applications, e.g., industrial machines, robots, rail vehicles, cars, cranes, aviation, electronics, and electrical engineering. The products used for this protection have to be simple in design and assembly and reliable in operation. For producing corrugated casing pipes and accessories, materials with a high mechanical strength and chemical resistance are used, including PA6 and PA12 polyamides, polyethylene (PE), PP, thermoplastic polyester (TPE), and thermoplastic polyurethane (PUR). The advantages of PP and PUR as impulse-loaded matrices of laminates were demonstrated in [17,18].

Conduits and hoses are secured by fittings, which should have a small bend radius. In addition, they have to withstand loadings from 90 to 850 N for every 100 m of length and the ability to work in the temperature range from −50 to +150 °C. In manipulators, robotics, automation, and moving machines, multipoint assemblies of conduits with various covers, connections, clamping elements, rotary handles, and cable glands are used. They can be mounted on the bases of robots and manipulators and have to be capable of rotating, articulating, and automatically adjusting the length by using springs.

In industrial drive systems of machines and devices, pneumatic systems are widely used as key elements in the automation of production processes. Pneumatic systems are currently the most ecological and economical media for industry, but they are used in less demanding processes. The complexity of designs and multiplicity of applications of pneumatic systems results mainly from the advantages of compressed air, which enables the generation of significant forces, and from a large assortment of valves [19,20,21] and pneumatic components. In industry, pneumatic installations for the distribution of compressed air are commonly made of PP-R polypropylene or PP-R polypropylene with an external aluminum layer and on fasteners, connected by welding with a temperature resistance up to 60 °C and a maximum pressure of 20 bar.

Smooth-wall PP pipes are widely used for gravity sewer systems and networks. Although pipes comprise only ~4% of the total cost of building a pipeline system, they are essential element of the system [22,23]. The pipes are subjected to complex loads, primarily mechanical stresses caused by static ground loads, and dynamic transport loads [24,25,26]. High-quality assurance of the pipes can conflict with financial considerations. Attempts at reducing the production cost (often without informing the consumer) through the excessive use of inorganic fillers and recycled plastics of unknown origin [6] illustrate the seriousness of the contradiction. A large number of competing manufacturers, replacement of traditional polyvinyl chloride and polyethylene with PP [4], a wide range of the raw materials used and a variety of manufactured pipes are the objective market trends, which make an optimal selection of the properties and design of pipes difficult.

A typical example is choosing between an “expensive” single-layer pipe made of a dense material and a “cheap” multilayer pipe with a lightweight foam core. Producers and consumers equally face this choice on a tight budget. Both the pipes have the same exterior and interior material. This makes it possible to ensure the same ring stiffness and flexibility properties and formally meet the same standards (the nominal pipe ring stiffness class (SN)). In turn, the tensile tests, whose results are traditionally used to control the quality of the material, can be very sensitive to changes in the composition and geometry of pipe layers.

The desire to optimize this choice provided an impetus for the development of the concept of this study. This concept includes a comparative study of the two types of pipes mentioned, differing in price, from the same manufacturer. Flexural and tensile tests on full-thickness specimens, analyses utilizing the finite-element method (FEM), and collating findings with the common knowledge will be performed. Analytical engineering estimates of the geometry of layers of a multilayer pipe will be proposed based on the required effective characteristics for the pipe, taking into account the characteristics of its layers. The implementation of the concept will achieve the following goal: to develop a prompt and simple method for determining the mechanical properties of industrial multilayer-extrusion pipes in relation to the geometry and properties of their layers and vice versa.

## 2. Materials and Methods

### 2.1. Materials and Making of Specimens

Three types of smooth-wall PP pipes for a gravity sewer network of nominal pipe ring stiffness class SN8 were considered in the work. The pipes were manufactured by SIA EVOPIPES, Jelgava, Latvia (their corresponding declared ring stiffness has to be ≥8 kN/m^2^).

Two types of the pipes were tested, namely:

- A RIGID MONO PP single-layer solid-wall pipe (designated as PPS), produced from PP material with a homogenous flexural modulus;
- RIGID MULTI PP pipes (designated as PPM) produced using three-layer technologies. The pipes had external (*e*) and internal (*i*) layers made of polypropylene with a high flexural modulus. The intermediate (*m*) layer was made of PP foam.

For tests on the *m*-layer of the PPM pipe, a RIGID MULTI PP pipe of greater diameter and, accordingly, a larger thickness of each layer, designated as 3PPM, was used. Its diameter was 315 mm, and this thickness was 11.5 ± 0.3 mm. The charakt of the pipes tested are indicated in Table 1.

**Figure 1:**
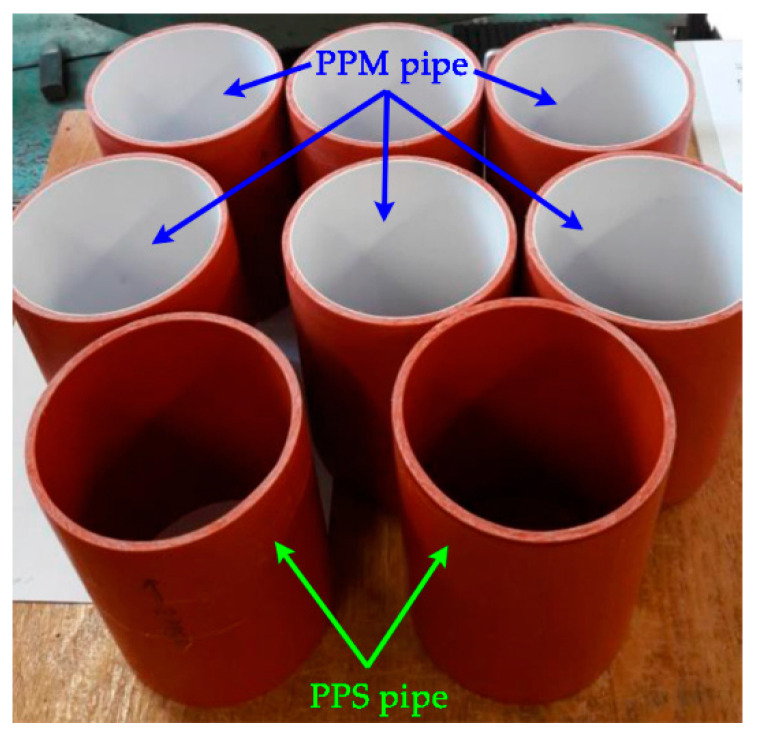
Pipe pieces.

The PPS and PPM pipes (Figure 1) were sawn into pieces of length *L* ≈ 153 mm and processed by sandpaper. They were used to determine the ring stiffness, flexibility, modulus and density.

**Figure 2:**
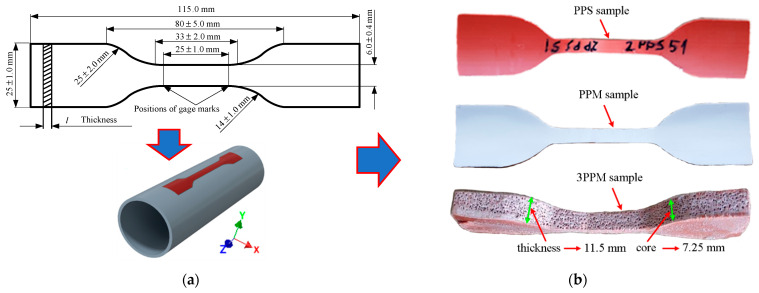
Preparation of tensile test specimens from pipes: (**a**) cutting technology of samples from pipes; (**b**) geometry of the samples tested.

For tensile tests, five sectors were used, and five strips were cut from the PPS and PPM pipes, as supplied, along the pipe axis according to [27]. Test specimens were manufactured with a cutting die from the PPS and PPM pipes. Several specimens were also cut from the 3PPM pipe (Figure 2).

The dimensions of the dumbbell-type tensile test specimens prepared were as follows: overall length 115 mm, length of the narrow parallel-side portion 40 mm, width 5.7 mm. To determine the properties of the *m*-layer, tensile test specimens were manufactured by sanding off the outer and inner solid layers of the dumbbell-type 3PPM specimens (such a specimen manufactured from thin PPM pipes with a 2 mm thickness foam layer was not possible). These specimens had the following dimensions: overall length 115 mm, length of the narrow parallel-side portion 40 mm, width 4.97 ± 0.02 mm, thickness 6.4 ± 0.5 mm. Cuboid specimens were sawn and sanded to determine the density of the foam layer of PPM and 3PPM specimens.

### 2.2. Mechanical Tests and Determination of Density

In accordance with the concept of the comparative study of two pipes from the same manufacturer, when conducting the ring stiffness and flexibility and tensile tests, the easier and promptly-run ring stiffness and tensile tests were used. The first test established a basis for the classification of sewer pipes as per International and European standards—their SN class had to be determined or confirmed. This depended directly on the pipe modulus in the loading scheme used. The second test (tensile) allowed us to draw information about the properties of the pipe-material (elastic modulus, ultimate strength, etc.)

**Figure 3:**
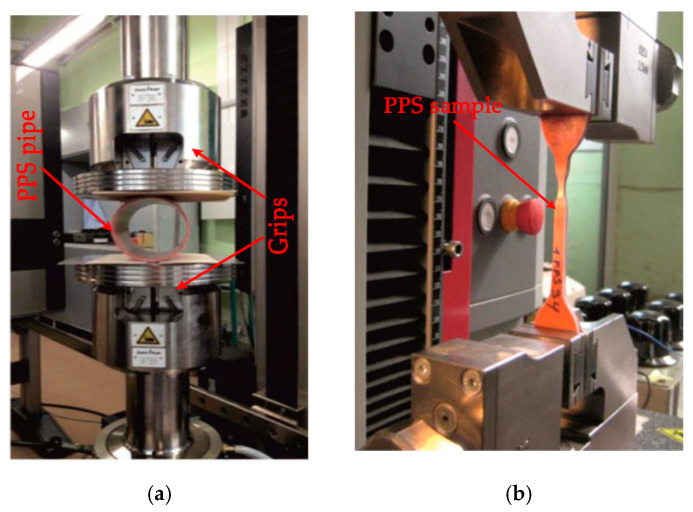
Testing of specimens: (**a**) initial stage of testing the ring stiffness and flexibility of a PPS sample; (**b**) tensile testing of a PPS specimen.

The ring stiffness and flexibility tests were carried out, according to [28,29] respectively, at 16–20 °C on a Zwick Z100TEW (ZwickRoell, Wroclaw, Poland) mechanical testing machine with a crosshead speed of 12.5 mm/min (Figure 3a). Deflections of specimens were found from grip displacements. Six specimens, cut from two sections of pipes, were tested for each type of PP pipes. Before testing, the specimens were conditioned at 14–20 °C for more than 40 h. In tests, the ring specimens were loaded until at least 30% deflection was reached or their fracturing occurred.

The tensile tests of 4.02 and 4.25 mm thick specimens manufactured from PPS and PPM pipes, respectively, were carried out, according to ISO 6259-1 and 6259-3, at 19–21 °C on a Zwick 2.5 mechanical testing machine with a crosshead speed of 100 mm/min (Figure 3b). The same speed was also used for 11.5 and 7.25 mm thick specimens, manufactured from 3PPM pipe and its intermediate foam layer, respectively (see Figure 2b). The elongation of the test pieces was found from grip displacements. Before tests, the specimens were conditioned at 17–21 °C for more than 40 h.

The density *ρ* was determined as the ratio of weight *m* to the volume *V* of a pipe/layer piece with a known geometry. Thus, the *m*-layer (PP foam) densities $\rho_{m}$ were determined from cuboid specimens cut from the layers of PPM and 3PPM pipes. These densities were used for a linear interpolation of the unknown tensile characteristics of the *m*-layer of PPM.

## 3. Approaches to the Issue and Numerical Simulations

### 3.1. Assumptions

The combination of high stiffness and good impact strength is caused by the highly crystalline structure of PP, providing the stiffness and the well-dispersed elastic phase, which is responsible for the impact strength of the pipe [30,31]. In deformation processes, a one-to-one relationship between stresses and strains is observed only at small strains. When the tensile stress *σ* reaches a certain limit value $\sigma_{0}$, called the yield point in tension, plastic strains arise. The plasticity criterion has the form:

(1) $$\sigma^{2}-\sigma_{0}^{2}=0$$

In the case of pure shear, the plasticity criterion for the tangential stress τ is

(2) $$\tau^{2}-k^{2}=0$$

where *k* is the yield stress in pure shear.

We assume that there exists a scalar function *f*, defined on the set of tensors $T_{2}^{S}$, called the flow function, and the yield condition has the form:

(3) f(σ)=0

For isotropic solids, *f* is a function of two $II_{S}$ and $III_{S}$ of the stress deviator *S*:

(4) $$f\left(II_{S},III_{S}\right)=0$$

The plasticization of ductile materials can be described using the criterion of maximum shear energy—the Huber—von Mises—Hencky criterion [32].

According to this criterion, the plastic transition of a material is determined by the level of shear energy. This criterion can be written in the form:

(5) $${\left(\sigma_{I}-\sigma_{II}\right)}^{2}+{\left(\sigma_{II}-\sigma_{III}\right)}^{2}+{\left(\sigma_{III}-\sigma_{I}\right)}^{2}=2\sigma_{0}^{2}$$

where $\sigma_{I}$, $\sigma_{II}$, and $\sigma_{III}$ are the principal stresses.

With $\varepsilon^{p}$ as the plastic strain, *k* as functional depending on the stress–strain state, and *T* as temperature, the yield criterion has the form:

(6) $$f\left(\sigma ,\;\varepsilon^{p},k,\;T\right)=0$$

The stress vector is defined as

(7) $$P=\underset{\Delta S\to 0}{\lim}\frac{\Delta P}{\Delta S}=\frac{dP}{dS}$$

where Δ*S* is an area element.

The displacement u has three components:

(8) $$u=u\left(u_{1},u_{2},u_{3}\right)$$

which are functions of coordinates and time:

(9) $$u_{i}=u_{i}\left(x,y,z,t\right)$$

The Euler strain tensor is expressed as

(10) $$e_{ij}=0.5\left(u_{ij}+u_{ji}-u_{mi}\cdot u_{mj}\right)$$

The tensor $e_{ij}=0.5\left(u_{ij}+u_{ji}\right)$ is called the tensor of small strains. We define the intensity of strains as

(11) $$\varepsilon_{e}=\sqrt{\frac{2}{3}\varepsilon_{ij}\cdot \varepsilon_{ji}}$$

The hypothesis about the component of the function *F* on the boundary of the region corresponding to the elastic state can be written as

(12) $$F=F\left(\sigma_{ij},\varepsilon_{ij}^{p},k\right)$$

where $\sigma_{ij}$ and $\varepsilon_{ij}^{p}$ are components of the stress and plastic strain tensors, respectively, and *k* is a functional dependent on the stress-strain state.

To determine the function *F* from Equation (12), the Huber—von Mises—Hencky criterion is used, in which the transition to the plastic state is determined by the deformation energy of the material, i.e., by the second invariant of the stress deviator. Thus, we have

(13) $$F=J_{2S}-\frac{1}{3}Y^{2}=\frac{1}{3}S_{ij}\cdot S_{ji}-\frac{1}{3}Y^{2}$$

where $J_{2S}$ and $S_{ij}$ are the stress tensor invariant and deviator, respectively; *Y* is the Johnson–Cook (J-C) model function, defined as

(14) $$Y=\left[A+B{\left(\varepsilon^{p}\right)}^{n}\right]\cdot \left(1+C\ln{\dot{\varepsilon }}_{*}^{p}\right)\cdot \left[1-{\left(T_{*}\right)}^{m}\right]$$

Here, *A*, *B*, *C*, *n,* and *m* are material constants; ${\dot{\varepsilon }}_{*}^{p}$ and $T_{*}$ are the normalized changes in the intensity of strains in terms of plastic strain rate and temperature.

### 3.2. Numerical Models

Numerical calculations were performed in the ABAQUS software by using the explicit method. Models of the samples produced were analyzed in accordance with the tests performed. The foam core of the 3PPM sample was developed as the three-dimensional foam model was reconstructed using the technical microtomography method [33]. The obtained results of the reconstruction were segmented using the local adaptive method of data thresholding. The volume of the volumetric model acquired in this way is approximated by a surface model (triangle mesh). The mesh of triangles was then processed to improve its quality, eliminate surface interference, and reduce the number of triangles. The surface grid thus prepared was converted to a three-dimensional “tetra” grid, allowing it to be loaded into the FEM simulation software.

**Figure 4:**
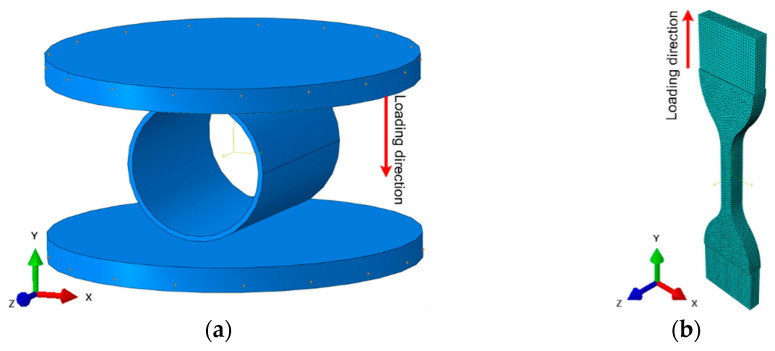
Examples of finite-element simulations in the ABAQUS code: (**a**) compression of a pipe; (**b**) extension of a specimen.

An example of the modeling work is shown in Figure 4a, where a pipe is located between two rigid jaws. The lower jaw is fixed, but the upper jaw compresses the pipe quasi-statically. Similarly, the tensile test was also modeled (Figure 4b).

For simulation purposes, the pipe and jaws were modeled as tetra-type and rigid objects, respectively. The time step was 0.01 s, and the total simulation time was 100 s. Tetra-type elements with a size of 0.5–2 mm were used, depending on the geometric arrangement. Additionally, the limit strain corresponding to the structure discontinuity was imposed.

It should be noted that in this case, the analyzed samples with a foam core have a porous structure and appropriate geometry, which was imported from computed tomography in the form of a “tetra” grid, and therefore, it was decided to use the tetra element (C3D10M). Due to the fine tuning of the numerical model and the application of the limit strain, the same elements and their sizes were assumed for the entire volume. Thanks to this, the element used or its size did not affect the discontinuity of the element structure.

In accordance with Equation (14), the constitutive J-C model was adopted in the form:

(15) $$\sigma_{y}=\left(A+B\varepsilon^{n}\right)\cdot \left[1+C\ln\left(\frac{\dot{\varepsilon }}{{\dot{\varepsilon }}_{0}}\right)\right]$$

as an elastic-viscoelastic model, where $\sigma_{y}$ is the equivalent stress, *ε* is the equivalent plastic strain, *A* is the yield stress of the material under reference conditions, *B* is the strain hardening constant, *n* is the strain hardening coefficient, *C* is the strengthening coefficient of the strain rate, $\dot{\varepsilon }$ is the ratio of the equivalent plastic strain rate, and ${\dot{\varepsilon }}_{0}$ is the reference strain rate.

The contact model was based on the “hard” contact relationship, with the friction coefficient set to 0.1. The boundary conditions were given such that the numerical model reproduced features of the real phenomenon as accurately as possible.

The material constants of the analyzed pipes are summarized in Table 2.

## 4. Results of Tests and Simulations

### 4.1. Ring Characteristics

In ring stiffness and flexibility tests, the load *F* (the radially applied force *F* per pipe length *L*)–deflection Δ*y* and the load–relative deflection $\frac{\Delta y}{\left(D-e\right)}$ diagrams were obtained, where D−e is the mean diameter or the mid-wall diameter. The initial, practically linear, section of the diagram (up to a 3% deformation of the internal diameter of pipe) can serve to determinate the pipe stiffness *PS*. In practice, the pipe stiffness was determined as the slope of secant of the initial section of the load—deflection curve:

(16) $$PS=\frac{F}{L\cdot \Delta \left(\Delta y\right)}$$

The ring stiffness $S_{R}$, based on analytical calculations of structural mechanics [32,34,35], was found to be:

(17) $$S_{R}=0.0187\cdot PS=\frac{E_{p}\cdot e^{3}}{12D^{2}}$$

**Figure 5:**
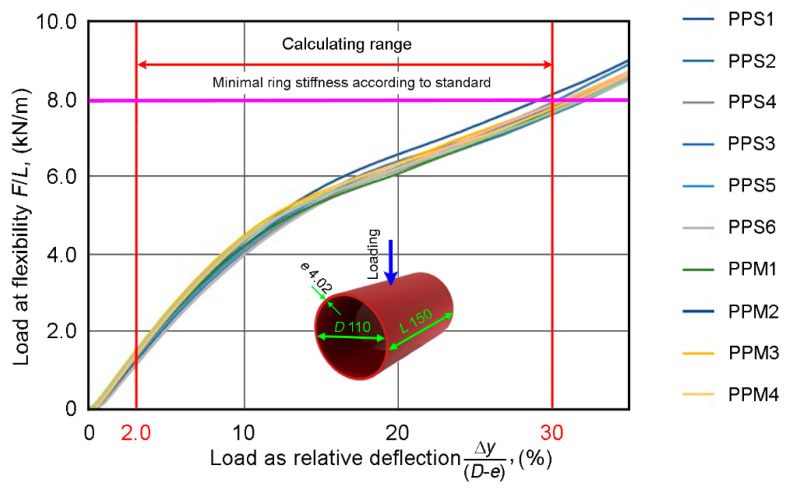
Ring stiffness and flexibility characteristics.

Relation (17) allows one to determine the pipe modulus $E_{p}$ taking into account the geometric parameters of pipe specimens. Because the ring stiffness classes declared were the same and the outer layers of both types of pipes, which determine the flexural properties, were also the same, the pipe modulus $E_{p}$ was expected to be insensitive to the difference in the structures of PPS and PPM pipes. The test diagrams (see Figure 5) of both types of pipes coincided, as expected, without any indication of pipe-structure changing from a single- to multilayer type. The results of ring flexibility tests up to a 30% deflection were trivial, and no cracking, delamination, or rapture occurred in either of the types of pipes. Moreover, the specimens tested did not fracture at all. The quantitative characteristics (with standard deviations and relative standard deviations (%) in brackets), calculated from the diagrams, are indicated in Table 3. A row with differences in characteristics (ratios of difference between the values of PPM and PPS to the value of PPS) is also added. The sum of relative standard deviations of ring stiffness and load at 30% flexibility of PPS and PPM exceeded the corresponding relative changes. This did not allow us to recognize the changes in the values as significant. Only pipe moduli demonstrated a slight difference over the sum of relative standard deviations. These percentage deviations amounted to 2.7% for the PPS sample and 4.2% for the PPM sample. The reason for this distinction was the different thicknesses *e* of the pipes studied—the elastic modulus depends on *e* to the third power (see Equation (17)).

As expected, we can conclude that the ring stiffness and flexibility tests of the two pipe types confirmed their declared SN class, did not reveal any difference, and did not allow us to choose between an “expensive” single-layer pipe made of a dense material and a “cheap” multilayer pipe with foam, as shown in Figure 5.

### 4.2. Ring Stiffness and Flexibility in Numerical Simulations

**Figure 6:**
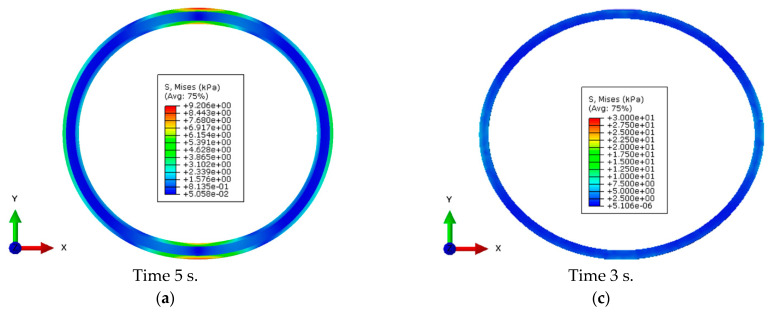

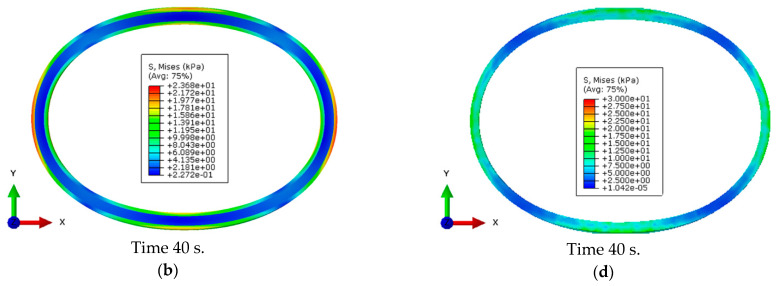
Numerical analysis of ring stiffness and load at 30% flexibility of the pipe investigated: (**a**,**b**) selected time steps in compressing the PPM sample; (**c**,**d**) selected time steps in compressing the PPS sample.

The numerical compression tests of PPS and PPM pipes were performed as described in Section 3.2. The compression conditions were identical to those in the pipe stiffness test. The results obtained are presented in Figure 6.

It can be seen that, within the same declared stiffness limits, the PPS pipe had lower stiffness than the PPM pipe. This is explained by the differences in their structure. The PPM pipe had a sandwich structure with a foam core, and therefore, it was more susceptible to deformation, as is seen from the distribution of stress values according to the Huber—von Mises hypothesis. The average stiffness of the PPM pipe was approximately 9.98 kPa, but that of the PPS pipe, 8.75 kPa. These results correspond with the data already known from literature [36,37].

### 4.3. Tensile Characteristics

**Figure 7:**
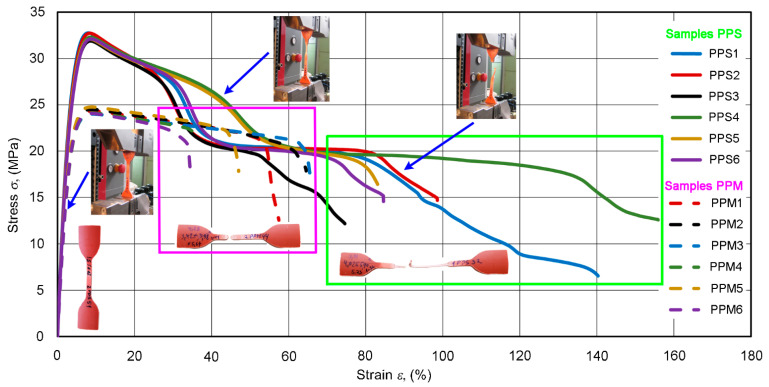
Stress–strain curves *σ*–*ε* of PPS and PPM specimens.

The tensile stress–strain diagrams of six PPS and six PPM specimens, shown in Figure 7, demonstrate the tensile behavior common to PP [2]. At first, the PPS and PPM diagrams showed an almost linear growth up to about 23 and 19 MPa, respectively. The modulus “falls” then decreased steeply to a zero value. At that time, the necking of specimens propagated along their entire length. The necking was more pronounced in the PPS specimen. As a result, the average elongations at break (fracture strains) were about 112% and 47% for the PPS and PPM specimens, respectively (less than 200%, according to standard [38]), but this elongation differed greatly (see Figure 6). This fact hindered us from taking this characteristic as the sought-for feature sensitive to changes in the structure from PPS to PPM.

**Figure 8:**
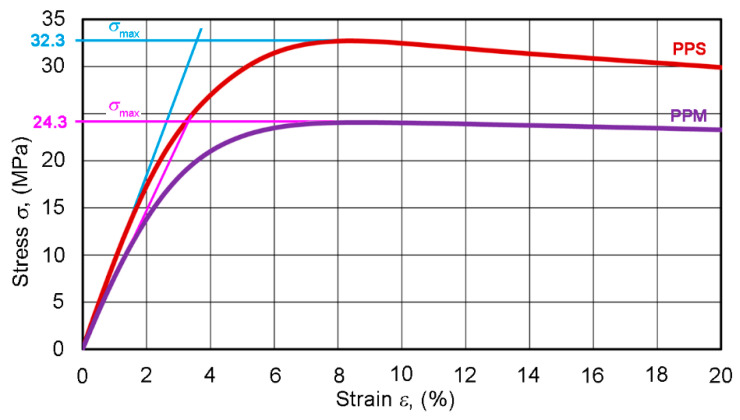
An enlarged view of the initial section of tensile stress—strain curves of typical PPS and PPM specimens. The inclined straight lines are secants at a 0.3–1.5% elongation, but the horizontal straight lines determine the value of *σ*_max_.

Let us highlight the initial sections of two typical PPS and PPM deformation curves (Figure 8). The enlarged view of the sections shows that, for each pipe type, the curve differs markedly. This contrasts with the ring test diagrams discussed above. The difference in the incline of the initial almost linear sections of the diagrams points to the potential importance of the tensile elasticity modulus *E* for studying the implementation of our concept. As the diagrams of both pipe types deviate from Hooke’s law (as is usual for many plastics), *E* was calculated as the slope of secant of the initial section of the stress–strain curve at a 0.3–1.5% variation in the strain *ε*.

In the section of varying curvature of the *σ*–*ε* curve, specimens deformed plastically. The ultimate tensile strength corresponds to the maximum stresses in Figure 7 and Figure 8 [39]. The easily detectable and stable *σ*_max_ points in the diagrams were also potentially important as characteristics sensitive to changes in the structure of composites.

The results of tensile tests are summarized in Table 4. Analyzing the important quantitative characteristics calculated from tensile tests—the tensile modulus *E* and the ultimate strength *σ_max_*, we arrived at the following conclusions.

These two characteristics noticeably sensed the difference between pipe structures. The sum of relative standard deviations was much smaller than the revealed relative change in the values of these characteristics.

In addition, when analyzing the standard deviation for specific parameters, it was found that, for the strain at break, its value was 42% for the PPS samples and 20% for the PPM samples. For the tensile modulus, it was 2.3% for the PPS samples and only 1.4% for the PPM samples. Slight differences were also recorded for the ultimate tensile strength—its value was 3.1% for the PPS samples and 1.2% for the PPM samples. Despite the greater difference (57.7%) in the mean value of the strain at break, its standard deviation was also quite high, implying that its actual value had a high dispersion (and could be dependent on other factors not considered in the current tests). Thus, this parameter is not reliable enough to optimize the selection of pipe materials and does not allow one to distinguish between the quality of constituent materials of pipes at the scope of tests in the engineering practice.

### 4.4. Numerical Tensile Characteristics

Numerical simulations of the stretching of samples made of PPS and PPM pipes were carried out employing the ABAQUS software, adopting the constitutional model described by Equation (15). The values of material constants were taken in accordance with Table 2, which reflect the results obtained from tensile strength tests (see Table 4).

**Figure 9:**
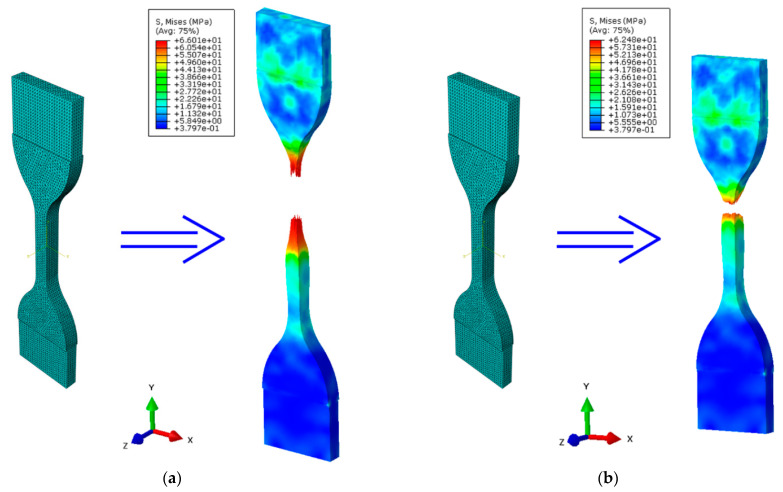
Selected numerical results from the tensile test of samples: (**a**) stress–von Mises distribution in PPS samples; (**b**) stress–von Mises distribution in PPM samples.

The results of numerical experiments were obtained in the form of stress contour lines based on the Huber–von Mises hypothesis. The results for the samples are summarized in Figure 9.

**Figure 10:**
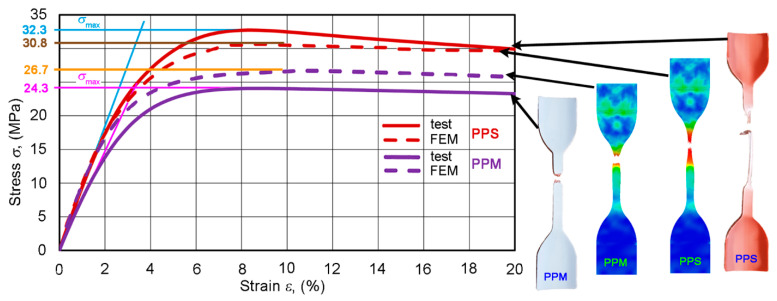
Comparison of FEM and experimental results in the tension of PPS and PPM pipe samples.

The results obtained show that the FEM data is slightly different, which is a consequence of the ideal initial boundary conditions assumed. For the PPS sample, the averaged ultimate tensile strength was 30.8 MPa (see Figure 9a), but for the PPM sample—26.7 MPa (see Figure 9b). Composite materials are viscous [40,41,42], which led to some dispersion of experimental results, which are presented in Figure 10.

As seen in Figure 10, the FEM data obtained is the result of a positive compliance of the fit of the material model with the outcomes of strength tests. The level of accordance of the results obtained is in the range of good matching (Δe ≤ 10%) [43].

### 4.5. Properties of the Layers

Based on manufacturer’s information, we assumed that the properties of both the *e*- and *i*-layer of PPM pipes were the same as those of single-layer PPS pipes. Thus, the density, tensile modulus, and ultimate strength of the PPS pipe specimen (Table 4) were used as the corresponding values of *e*- and *i*-layers. The average thicknesses $e_{e}$ and $e_{i}$ of these layers of the PPM pipe were measured by an optical microscope in many locations of different specimens of each type, and it was found that $e_{e}=1.08\;\pm \;0.10,$ and $e_{i}=0.81\;\pm \;0.09$ mm. According to this data, the corresponding volume fraction of the layers in the PPM pipe were $v_{e}=0.266$ and $v_{i}=0.189$. The average thickness of the *m*-layer of the PPM pipe was $e_{m}$ = 2.3 ± 0.2 mm, from which it followed that $v_{m}=0.54$, i.e.,

(18) $$v_{m}+v_{e}+v_{i}=1$$

When trying to experimentally determine the tensile properties of the *m*-layer of PPM pipes, it turned out that it was too thin to make dumbbell-type specimens. On the contrary, the *m*-layer of 3PPM pipes was thick enough for this purpose, though it had larger voids and a lower density than the *m*-layer of PPM and could not be used to experimentally determine the tensile properties of the denser foam of PPM pipes. Therefore, we assumed that a linear interpolation of the properties of *m*-layers can be used for approximation of the unknown characteristics of PPM pipes.

Let us consider the tensile modulus of the *m*-layer as a linearly increasing function of foam density $\rho_{m}.$ The unknown tensile modulus $E_{1m}$ of the *m*-layer of PPM will be between two known ones: $E_{2m}$ and $E_{3m}$ at the lowest density $\rho_{2m}$ and the highest density $\rho_{m}$, defined as $\rho_{3}$, respectively. Here, the homogeneous material of PPS corresponds to a “foam” with a zero-void fraction.

The interpolation formula obtained for calculating the tensile modulus of the *m*-layer of PPM is

(19) $$E_{1m}=E_{2m}+\left(\rho_{1m}-\rho_{2m}\right)\cdot \frac{E_{3m}-E_{2m}}{\rho_{3}-\rho_{2m}}$$

where $E_{1m},$ $E_{2m},$ and $E_{3m}$ are the tensile moduli of PPM, 3PPM, and PPS pipes, respectively; $\rho_{1m}$, $\rho_{2m}$, and $\rho_{3}$ are the densities of PPM, 3PPM, and PPS pipes, respectively.

In the same way, the ultimate strength of the PPM pipe can be interpolated if $E_{1m}$ is replaced everywhere in Equation (19) in the following way:

(20) $$\sigma_{1m_{max}}=\sigma_{2m_{max}}+\left(\rho_{1m}-\rho_{2m}\right)\cdot \frac{\sigma_{3m_{max}}-\sigma_{2m_{max}}}{\rho_{3}-\rho_{2m}}$$

where $\sigma_{1m_{max}}$ is the ultimate strength of the PPM pipe, $\sigma_{2m_{max}}$ is the ultimate strength of the 3PPM pipe, and $\sigma_{3m_{max}}$ is the ultimate strength of the PPS pipe.

Thus, the interpolation formulas allow anyone to estimate the missing data of the foam layer of PPM pipes using the experimental characteristics of the foam layer of 3PPM pipes. All experimental and interpolated (are without a standard deviation) *m*-layer foam characteristics for the PPM and 3PPM pipes are summarized in Table 5. These results do not differ significantly from the results found by other researchers [44,45,46].

### 4.6. Recommendations for Engineering Assessments

The objects of this study are quite simple—multilayer pipe consisting of three isotropic layers, where the intermediate foam layer is more compliant than the internal and external layers. The density and tensile properties of the last two layers are equal to that of the single-layer (PPS) pipe material. All three layers are extruded simultaneously from one basic PP component, and therefore, it is expected that they deform equally at tension up to fracture. To form engineering assessments, let us use the simplest linearly elastic approximation (σ=Eε) and the rule-of-mixtures (RoM).

Since the stress—strain diagrams of PPS or PPM specimens are quasi-linear only at the initial interval of tension, let us replace the gradually-bending initial section of the diagram with a secant (blue and pink line) up the ultimate strength $\sigma_{max}$ (Figure 8). We then obtain simple analytical expressions for the effective density *ρ* and tensile modulus *E* as functions of layer properties in the form:

(21) $$\rho =\rho_{e}v_{e}+\rho_{i}v_{i}+\rho_{m}v_{m}$$

(22) $$E=E_{e}v_{e}+E_{i}v_{i}+E_{m}v_{m}=E_{e}\left(v_{e}+v_{i}\right)+E_{m}v_{m}$$

Let us assume that the real PPM material reaches its ultimate strength $\sigma_{max}$ when the stiffest *e*- and *i*-layers reach their ultimate strength $\sigma_{e_{max}}$, as in the case of deformation of a linear-elastic material. Using the effective stress:

(23) $$\sigma =\sigma_{e}v_{e}+\sigma_{i}v_{i}+\sigma_{m}v_{m}=\sigma_{e}\left(v_{e}+v_{i}\right)+\sigma_{m}v_{m}$$

and the ultimate-strength-to-modulus ratio for equally deformed layers:

(24) $$\;\frac{\sigma_{m}}{\sigma_{e_{max}}}=\frac{E_{m}}{E_{e}}$$

we then obtain a simple analytical expression for the effective ultimate strength of PPM:

(25) $$\sigma_{max}=\sigma_{e_{max}}\left(v_{e}+v_{i}+\frac{E_{m}}{E_{e}}v_{m}\right)$$

All that remains is to compare the experimental results with calculations by the formulas and to clarify if this simple model can be applied to the objects under study. This is provided in Table 6. It can be seen that the expected effective data density shows the coincidence of experimental and calculation results. The experimental value of the effective tensile modulus coincides (with a relative difference below 3%) with the calculated one, albeit interpolation results for the modulus of *m*-layer foam were used in Equation (22). This fact confirms the validity of using the interpolation approach and the chosen model for the materials studied. The ultimate tensile strength of PPM specimens differed more markedly because of the obvious inconsistency between the plastic flow of PP and the simple linearly elastic approximation. Nevertheless, in our opinion, the relative difference observed (ca. 12%) is still acceptable when using the simplified approach proposed.

Let us consider an important situation where the elastic moduli of PPM layers $E_{e}=E_{i}$ and $E_{m}$ and the expected effective modulus *E* are known in advance, and it is necessary to determine the thickness $e_{m}$ of the foam core. Based on the obvious fact that the volume $v_{m}$ fraction of the *m*-layer is the ratio of its cross-sectional area to that of the entire pipe and on geometric considerations, the volume fraction $v_{m}$ of the *m*-layer can be expressed as

(26) $$v_{m}=\frac{{\left(0.5D-e_{e}\right)}^{2}-{\left(0.5D-e+e_{i}\right)}^{2}}{{\left(0.5D\right)}^{2}-{\left(0.5D-e\right)}^{2}}=\frac{e_{m}D_{m_{mean}}}{eD_{m_{mean}}}$$

where $e_{m}=e-e_{e}-e_{i}$, $D_{mean}=D-e$ and $D_{m_{mean}}=D-e-e_{e}+e_{i}$ are the thickness of the *m*-layer, the mean diameter of the pipe, and the pipe diameter in the middle of the *m*-layer, respectively. Expressing $v_{m}$ from Equation (22) and inserting it in Equation (26), we then get the expression connecting the mentioned geometric parameters with expected tensile moduli as

(27) $$\frac{e_{m}D_{m_{mean}}}{eD_{mean}}=\frac{E_{e}-E}{E_{e}-E_{m}}$$

Thus, Equations (22), (25), and (27) are proposed for prompt and simple determination of the characteristics of single- or multilayer PP pipes for engineering calculations. These equations ensure an acceptable level of experimentally validated accuracy.

## 5. Conclusions

This work is addressed to manufacturers and users of extruded multilayer polypropylene pipes, who are interested in engineering evaluations of their mechanical properties. Despite the noticeable inelastic behavior of polypropylene, it was shown that the well-known analytical formulas of linear elastic approximation allow one, with acceptable accuracy, to promptly and simply determine the effective mechanical properties of multilayer pipes. Their properties depend on the thickness of the pipe and of each its layers. The results of the work allowed us to draw the following conclusions:

○ The smooth-wall single- and multilayer (with a foam core) polypropylene pipes manufactured at SIA EVOPIPES, with a nominal/external pipe diameter of 110 mm, demonstrated an experimental conformity to the same nominal class SN8 (in ring stiffness and flexibility tests) but exhibited markedly different tensile properties (the modulus and ultimate strength) of their materials;
○ The use of simple analytical formulas of linear interpolation, linear elastic approximation, and rule of mixtures (instead of using a more rigorous time-consuming approach) makes it possible to predict, with sufficient accuracy, the effective tensile properties of a multilayer pipe material based on the experimental data for each layer. The formulas mentioned can be used for estimating any unknown characteristic of layers and of the entire pipe from other known pipes and pipe geometry;
○ An expression connecting the multilayer PP pipe permissible thickness and relative position of the foam core with the expected tensile moduli of the entire pipe, and each layer can be applied to planning the pipe production process in order to minimize production costs by controlling the changes in pipe properties.

Judging from the results of FEM calculations, the simplified model (J-C) proposed correctly corresponded to the material characteristics determined in strength tests. The discrepancies between FEM and experimental results for the ring stiffness were 10.0% for the PPM pipes and 4.0% for PPS pipes, respectively. As regards the tensile characteristics, the difference was 4.6% for the PPS pipes and 9.0% for the PPM pipes. Taking into account the relative standard deviation for the experimental results, it should be emphasized that the results from FEM are very well correlated for PPS samples. Slightly worse results were obtained for the PPM samples, but this is the result of adopting the foam material data obtained from interpolation for multilayer pipes. Assuming that the ultimate tensile strength of PPM specimens differed more (see Table 6) and these results were adopted into the numerical model, the differences are greater.

These results can be considered suitable, as these differences were caused by many variables [47,48,49], as well as the results of interpolation of *m*-layer foam.

## Figures and Tables

**Table 1:** Types of tested pipes.

Designation	Diameter *D* (mm)	Declared Wall Thickness *e_min_* (mm)	Measured Wall Thickness *e* (mm)
PPS	110	3.8	4.02 ± 0.06
PPM	110	3.8	4.25 ± 0.07

**Table 2:** Material properties of pipes.

Designation	*ρ* (kg/m^3^)	*E* (MPa)	*v* (-)	*A* (MPa)	*B* (MPa)	*n* (-)
PPS	880	840	0.42	32	30	0.2
PPM	710	720	0.40	25	26	0.1
Foam core	700	620	0.03	-	-	-

*ρ*—Density, *E*—Young’s modulus, *v*—Poisson’s ratio.

**Table 3:** Test results for the ring stiffness and flexibility of PPS and PPM pipes with a nominal/external diameter *D* = 110 mm.

Designation	*e* (mm)	*L* (mm)	*S_R_* (kN/m^2^)	*E_p_* (GPa)	*F*/*L* (kN/m)
PPS	4.02 ± 0.06	153.50 ± 0.83	8.4 ± 0.3 (3.1)	1.84 ± 0.05 (2.7)	7.8 ± 0.2 (2.3)
PPM	4.25 ± 0.07	153.44 ± 1.05	8.9 ± 0.3 (3.3)	1.66 ± 0.07 (4.2)	7.9 ± 0.1 (1.3)
Differences, (%)	-	-	6.0	−10.0	0.4

*e*—thickness, *L* length, *S_R_*—ring stiffness, *E_p_*—pipe modulus, *F*/*L*—load at 30% flexibility (RF30), in brackets, the relative standard deviation (%) is given.

**Table 4:** Results of tensile tests on PPS and PPM specimens, with standard deviations in absolute values.

Parameters	*e* (mm)	*b* (mm)	*ρ* (g/cm^3^)	*ε_b_* (%)	*E* (MPa)	*σ_max_* (MPa)
PPS	3.93 ± 0.08	5.70 ± 0.09	0.9 ± 0.01	112.0 ± 47.0	880 ± 0.02	32.3 ± 1.0
PPM	4.16 ± 0.11	5.70 ± 0.05	0.7 ± 0.02	47.2 ± 9.6	720 ± 0.01	24.3 ± 0.3
Differences (%)	-	-	−22.2	−57.7	−18.8	−24.7

*e*—thickness, *b* width, *ρ*—density, *ε_b_*—strain at break, *E*—tensile modulus, *σ_max_*—ultimate tensile strength.

**Table 5:** Experimental and interpolated properties of the *m*-layer foam for different pipes.

Designation	Thickness *e_m_* (mm)	Density *ρ_m_* (g/cm^3^)	Tensile Modulus *E_m_* (MPa)	Ultimate Tensile Strength $\sigma_{m_{max}}$ (MPa)
PPM	2.3 ± 0.2	0.700 ± 0.020	620 (interpolation)	20.4 (interpolation)
3PPM	6.4 ± 0.5	0.614 ± 0.004	510 ± 0.03	15.3 ± 0.7

**Table 6:** Experimental and estimated properties of a PPM pipe and its layers.

Parameters and Characteristics	Thickness *e* (mm)	Layer Volume Fraction *v* (-)	Density *ρ_m_* (g/cm^3^)	Tensile Modulus *E_m_* (MPa)	Ultimate Tensile Strength $\sigma_{m_{max}}$ (MPa)
*e*-layer experiment	1.08 ± 0.10	0.266	0.90 ± 0.01	880 ± 0.02	32 ± 1.0
*i*-layer experiment	0.81 ± 0.09	0.189	0.90 ± 0.01	880 ± 0.02	32 ± 1.0
*m*-layer experiment	2.30 ± 0.20	0.545	0.70 ± 0.02	620 (interpolation)	not required
PPM experiment	4.20 ± 0.10	1.000	0.79 ± 0.02	720 ± 0.01	24.3 ± 0.3
PPM modeling	-	-	0.79	740	27.1
Formula	-	-	(21)	(22)	(25)

## Data Availability

Data sharing is not applicable to this article.

## References

[1] Spitael P., Macosko C.M. (2004). Strain hardening in polypropylenes and its role in extrusion foaming. Polym. Eng. Sci..

[2] Sara L., Abdelkhalek L., M’hamed C., Abdellah C., Ahmed D. (2015). Gravimetric, mechanical and chemical characterization of different materials used in sewers systems: Polyvinyl chloride (PVC), polypropylene (PP) and high density polyethylene (HDPE), aged in sulfuric acid at 60 °C. Res. Inven. Int. J. Eng. Sci..

[3] Weon J.-I. (2010). Effects of thermal ageing on mechanical and thermal behaviors of linear low density polyethylene pipe. Polym. Degrad. Stab..

[4] Genis A.V. (2017). Analysis of the global and Russian markets of polypropylene and of its main consumption areas. Russ. J. Gen. Chem..

[5] De La Fuente A., Pons O., Josa A., Aguado A. (2016). Multi-criteria decision making in the sustainability assessment of sewerage pipe systems. J. Clean. Prod..

[6] Wierzbicki Ł., Szymiczek M. (2012). Mechanical and chemical properties of sewage pipes. Arch. Mater. Sci. Eng..

[7] Mazurkiewicz ł., Małachowski J., Damaziak K., Tomaszewski M. (2018). Evaluation of the response of fibre reinforced composite repair of steel pipeline subjected to puncture from excavator tooth. Compos. Struct..

[8] Mazurkiewicz ł., Małachowski J., Tomaszewski M., Baranowski P., Yukhymets P. (2018). Performance of steel pipe reinforced with composite sleave. Compos. Struct..

[9] Zouhar M., Vallet L., Hutar P., Náhlik L. (2011). Life time estimation of the multilayer plastic pipes. Key Eng. Mat..

[10] Farshad M. (2005). Determination of the long-term hydrostatic strength of multilayer pipes. Polym. Test..

[11] Naguib H.E., Park C.B., Reichelt N. (2003). Fundamental foaming mechanisms governing the volume expansion of extruded polypropylene foams. J. Appl. Polym. Sci..

[12] Naguib H.E., Park C.B., Lee P.C., Xu D. (2006). A study on the foaming behaviors of PP resins with talc as nucleating agent. J. Polym. Eng..

[13] Reichelt N., Stadlbauer M., Folland R., Park C.B., Wang J. (2003). PP-blends with tailored foamability and mechanical properties. Cell. Polym..

[14] Yu K., Morozov E.V., Ashraf M.A., Shankar K. (2017). A review of the design and analysis of reinforced thermoplastic pipes for offshore applications. J. Reinf. Plast. Compos..

[15] Ek C.-G., Liedauer S., Mcgoldrick J., Ruemer F. (2005). Polypropylene Compositions Especially for Pipes. U.S. Patent.

[16] Hesse A., Lindstroem T., Ek C.-G., Rydin C., Hansen A. (2008). Singlelayer and Multilayer Polyolefin Foam Pipes. U.S. Patent.

[17] Bocian M., Jamroziak K., Kulisiewicz M., Pach J., Pyka D., Papadrakakis M., Fragiadakis M., Papadimitriou C. (2020). Numerical study of dynamic properties of a selected material layer of bul-letproof shields. EURODYN 2020: XI International Conference on Structural Dynamics.

[18] Mayer P., Pyka D., Jamroziak K., Pach J., Bocian M. (2017). Experimental and numerical studies on ballistic laminates on the polyethylene and polypropylene matrix. J. Mech..

[19] Blasiak S., Takosoglu J.E., Laski P.A. (2014). Optimizing the flow rate in a pneumatic directional control valve. Eng. Mech..

[20] Takosoglu J. (2017). Experimental research of flow servo-valve. Proceedings of the EPJ Web of Conferences.

[21] Blasiak S., Takosoglu J.E., Laski P.A., Pietrala D.S., Zwierzchowski J., Bracha G., Nowakowski L., Blasik M. (2017). Experimental and simulation flow rate analysis of the 3/2 directional pneumatic valve. Proceedings of the EPJ Web of Conferences.

[22] Li W., Dong B., Yang Z., Xu J., Chen Q., Li H., Xing F., Jiang Z. (2018). Recent advances in intrinsic self-Healing cementitious materials. Adv. Mater..

[23] Makris K.F., Langeveld J., Clemens F.H.L.R. (2020). A review on the durability of PVC sewer pipes: Research vs. practice. Struct. Infrastruct. Eng..

[24] Alzabeebee S., Chapman D.N., Faramarzi A. (2018). A comparative study of the response of buried pipes under static and moving loads. Transp. Geotech..

[25] Yang K., Xue B., Fang H., Du X., Li B., Chen J. (2021). Mechanical sensitivity analysis of pipe-liner composite structure under multi-field coupling. Structures.

[26] Fang H., Tan P., Du X., Li B., Yang K., Zhang Y. (2020). Mechanical response of buried HDPE double-wall corrugated pipe under traffic-sewage coupling load. Tunn. Undergr. Sp. Technol..

[27] ISO 6259-1 (2015). Thermoplastics Pipes—Determination of Tensile Properties—Part 1: General Test Method.

[28] ASTM D2412-21 (2021). Standard Test Method for Determination of External Loading Characteristics of Plastic Pipe by Parallel-Plate Loading.

[29] ISO 13968 (2008). Plastics Piping and Ducting Systems—Thermoplastics Pipes—Determination of Ring Flexibility.

[30] Pi L., Nie M., Wang Q. (2019). Crystalline composition and morphology in isotactic polypropylene pipe under combining effects of rotation extrusion and fibril β-nucleating agent. J. Vinyl. Addit. Technol..

[31] Ji H., Zhou X., Chen X., Zhao H., Wang Y., Zhu H., Wang Y., Zhu H., Ma Y., Xie L. (2020). Deformation-induced crystallization behavior of isotactic polypropylene sheets containing a β-nucleating agent under solid-state stretching. Polymers.

[32] Barsanescu P.D., Comanici A.M. (2017). von Mises hypothesis revised. Acta Mech..

[33] Ziółkowski G., Pach J., Pyka D., Kurzynowski T., Jamroziak K. (2020). X-ray computed tomography for the development of ballistic composite. Materials.

[34] Xi S.Z., Ying W., Wei J.P. (2019). Reliability analysis of buried polyethylene pipeline subject to traffic loads. Adv. Mech. Eng..

[35] Park J.-S., Hong W.-H., Lee W., Park J.-H., Yoon S.-J. (2014). Pipe stiffness prediction of buried GFRP flexible pipe. Polym. Polym. Compos..

[36] de Leeuw L.W., Martin G., Milewski H., Dietz M.S., Diambra A. (2020). Polypropylene pipe interface strength on marine sandy soils with varying coarse fraction. Proc. Inst. Civ. Eng. Geotech. Eng..

[37] Thörnblom K., Nilsson S.F., Sällberg S.-E., Bergström G. (2007). Durability of Non-Pressure Polypropylene Pipe Materials.

[38] ISO 6259-3 (2015). Thermoplastics Pipes—Determination of Tensile Properties—Part 3: Polyolefin Pipes.

[39] ASTM D638-14 (2014). Standard Test Method for Tensile Properties of Plastics.

[40] Altan M., Cankaya N. (2018). Thermoplastic foams: Processing, manufacturing, and vharacterization. Recent Research in Polymerization.

[41] Sadik T., Pillon C., Carrot C., Ruiz J.A.L., Vincent M., Billon N. (2017). Polypropylene structuralfoams: Measurementsof the core, skin, andoverall mechanicalproperties withevaluation ofpredictive models. J. Cell. Plast..

[42] Albooyeh A., Eskandarzadeh S., Mousavi A. (2019). Influence of different foaming conditions on the mechanical, physical, and structural properties of polypropylene foam. Mech. Adv. Compos. Struct..

[43] Pyka D. (2020). Dynamic Identification of Sandwich Composite Panels Subjected to Shock Loads. Ph.D. Thesis.

[44] Lee S., Zhu L., Maia J. (2015). The effect of strain-hardening on the morphology and mechanical anddielectric properties of multi-layered PP foam/PPfilm. Polymer.

[45] (2018). SystemSpecification-for PROGEF Standard Piping Systems in Polypropylene (PP).

[46] Maheo L., Viot P. (2013). Impact on multi-layered polypropylene foams. Int. J. Impact Eng..

[47] Domaneschi M. (2012). Experimental and numerical study of standard impact tests on polypropylene pipes with brittle behaviour. Proc. Inst. Mech. Eng. Part B J. Eng. Manuf..

[48] Rumianek P., Dobosz T., Nowak R., Dziewit P., Aromiński A. (2021). Static mechanical properties of expanded polypropylene crushable foam. Materials.

[49] Barsoum I., Almansoori H., Almazroueiand A.A., Gunister E. (2020). Fracture mechanics testing andcrack propagation modelling inpolypropylene pipes. Int. J. Struct. Integr..

